# IgA Vasculitis Followed by IgA Nephropathy Without an Identifiable Trigger: The Same Disease or a Spectrum of Related Conditions?

**DOI:** 10.7759/cureus.45639

**Published:** 2023-09-20

**Authors:** Rebecca Lapides, Juan Crespo-Quezada, Teena Thomas, Felipe Carmona Pires, Gagandeep Chera

**Affiliations:** 1 Department of Internal Medicine, Robert Larner, M.D. College of Medicine at the University of Vermont, Burlington, USA; 2 Department of Medical Education, Robert Larner, M.D. College of Medicine at the University of Vermont, Burlington, USA; 3 Department of Internal Medicine, Connecticut Institute for Communities, Danbury, USA; 4 Department of Internal Medicine, Danbury Hospital, Danbury, USA

**Keywords:** iga mediated vasculitis, palpable purpura, petechial rash, iga nephropathy, henoch-schönlein purpura (iga vasculitis)

## Abstract

IgA vasculitis and IgA nephropathy are characterized by IgA deposition in blood vessels and glomerular mesangium, respectively. The former is far more common in the pediatric population, while the latter presents more often in adulthood. A consensus regarding whether these two conditions are manifestations of the same disease that occurs on a spectrum has not yet been reached, and, to our knowledge, no clinical trials to address this question have been conducted. Here, we describe a 27-year-old patient who presented to the emergency department multiple times before being diagnosed with IgA vasculitis with no identifiable trigger and soon after developed IgA nephropathy. This case highlights the importance of ruling out these conditions, especially IgA vasculitis, in adults presenting with a petechial rash, but also the need for studies that investigate whether and how these conditions are related so that patients can be appropriately diagnosed and treated as efficiently as possible.

## Introduction

IgA vasculitis and IgA nephropathy are characterized by IgA deposition in blood vessels and glomerular mesangium, respectively [1,2]. IgA vasculitis is more common in the pediatric population, contributing to up to 45% of cases of vasculitis in children. IgA vasculitis is more rarely seen in the adult population, with an incidence of around 1.4-5.1 per 100,000 individuals [3]. Common clinical manifestations occur often after an identifiable trigger and include cutaneous purpura (commonly palpable or globs) or petechiae with lower limb predominance, arthralgia, GI symptoms such as abdominal pain, nausea, vomiting, renal involvement (glomerulonephritis), and rare cases of pulmonary involvement (hemorrhage) [4,5]. Various triggers have been reported, but usually, IgA vasculitis is preceded by a viral infection [1].

IgA nephropathy presents more often in adulthood, usually with macroscopic hematuria [2]. Existing research may suggest that IgA nephropathy and IgA vasculitis with renal involvement could be two clinical manifestations of the same disease that occurs on a spectrum and varies in symptomatic presentations and prognosis, although a definitive consensus has not yet been reached. Further, studies that include patients with IgA vasculitis or IgA nephropathy and follow them for a long enough period of time to make conclusions about whether these conditions are truly related are extremely limited. Existing fairly comprehensive reviews on this topic tend to suggest that these are two clinical manifestations of a common, underlying disease; however, clinical studies that can confirm this hypothesis are sorely lacking [6,7]. There are numerous case reports that describe patients with each of these conditions. There have also been some cases reported of patients developing both conditions [8]. However, it is still very rare that IgA vasculitis presents in adulthood and is followed by the development of IgA nephropathy. Thus, it is important to highlight these cases when they are encountered so that a more definitive understanding of these conditions and approach to treatment can be reached. Here, we present a 27-year-old female patient who presented to the emergency department and was eventually diagnosed with IgA vasculitis and subsequently developed IgA nephropathy.

## Case presentation

The patient is a 27-year-old female who initially presented to the emergency department with a rash and swelling of the bilateral lower extremities after consuming shellfish the night before. She denied fevers, chills, and pruritus. An allergic reaction was suspected, and she was given methylprednisolone, diphenhydramine, and famotidine in the emergency department and was discharged home on prednisone. Two days later, the patient returned to the emergency department concerned because the rash was migrating proximally, now present on the bilateral upper and lower extremities. Additionally, the rash was accompanied by diffuse myalgias in the bilateral lower extremities, worse on the left side.

**Figure 1:**
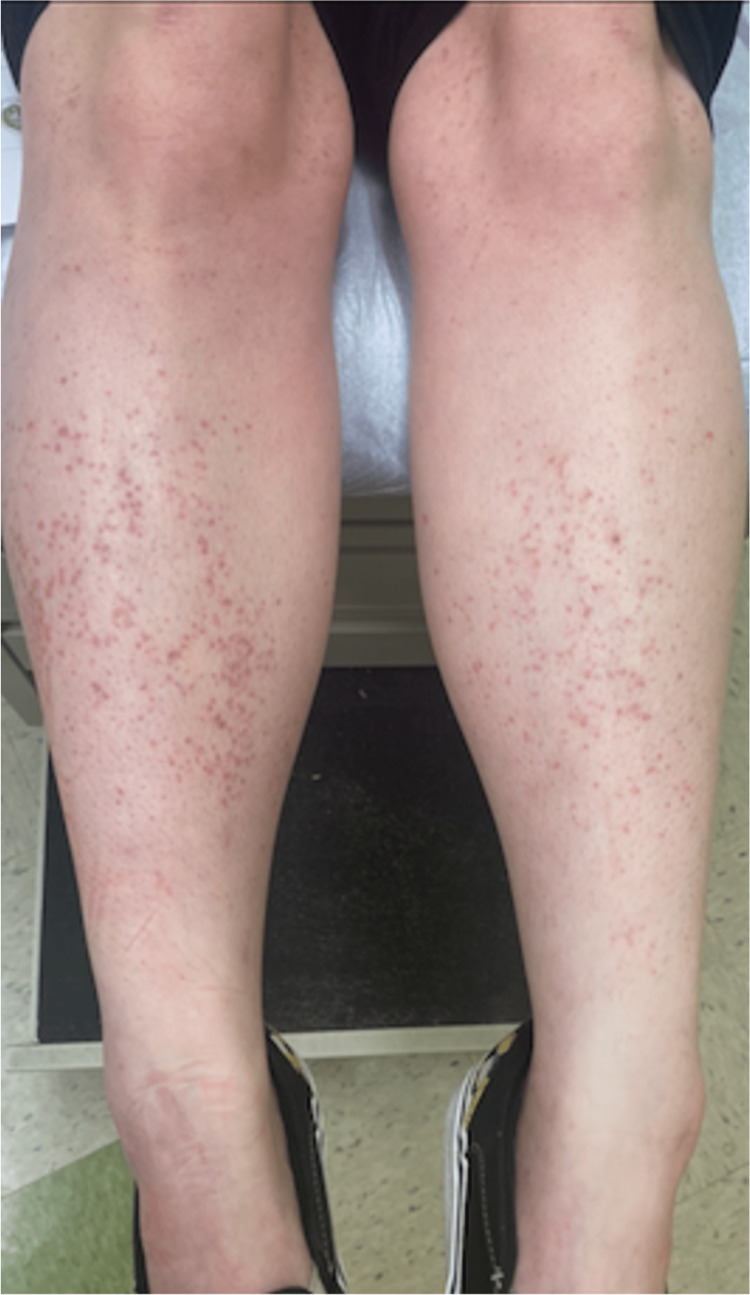
Bilateral lower extremity petechiae extending from the ankles to the distal thighs

On physical examination, vital signs were unremarkable. A scattered petechial rash was present in patches on the bilateral lower extremities extending from the ankles to the distal thighs (Figure 1).

The lesions were a red/purple color, macular, non-blanching, and non-tender to palpation.

The patient continued taking prednisone, which the lesions responded well to, and began tapering the prednisone over the following week. Nine days later, she returned to the emergency department complaining of sudden onset generalized abdominal pain radiating to the chest with nausea and vomiting. She had finished the prednisone taper the day prior and noticed the rash had returned and worsened with the subsequent development of abdominal pain. At that time, the patient had a white blood cell count of 19.5 with a left shift, elevated D-dimer at 840 ng/mL, albumin of 3.1 g/dL, and CRP at 3.9 mg/L. Urinalysis showed 2+ protein. Aerobic urine culture was done twice and both were negative. The protein-to-creatinine ratio was elevated at 5.633 mg/g, 24-hour urine microalbumin was 3,989 mg, and 24-hour urine protein was 6,900 mg. The infectious workup was negative for COVID-19, urinary tract infection, pneumonia, Lyme disease, chlamydia, gonorrhea, trichomonas, and hepatitis B and C. A CT angiography ruled out pulmonary embolism. CT abdomen and pelvis with contrast showed a right ovarian cyst, a distended stomach, and a thick-walled bladder.

**Figure 2:**
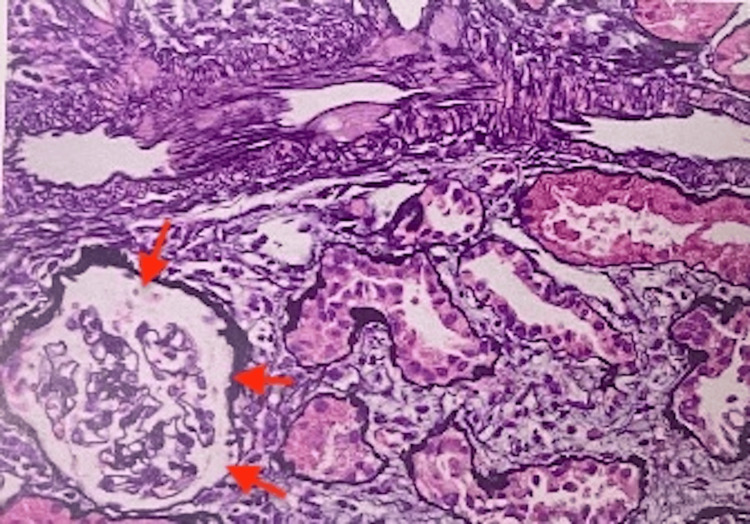
Kidney biopsy demonstrating intimal fibrosis (red arrows)

**Figure 3:**
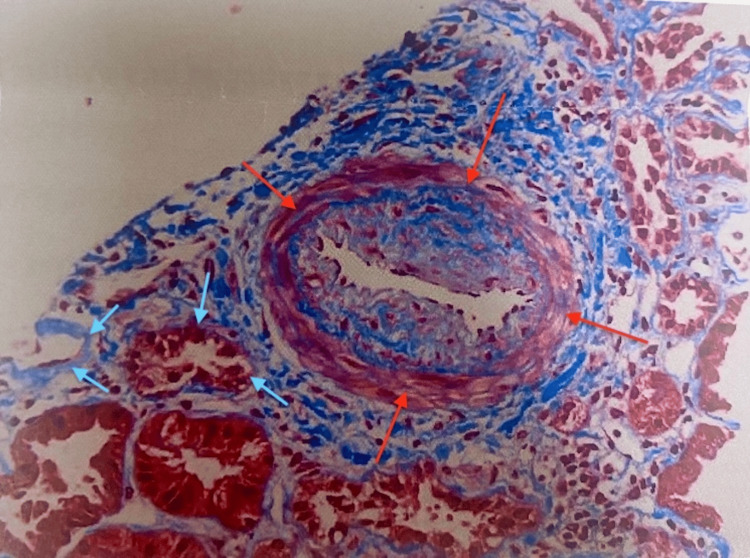
Kidney biopsy demonstrating interstitial fibrosis (red arrows) and tubular atrophy (blue arrows)

**Figure 4:**
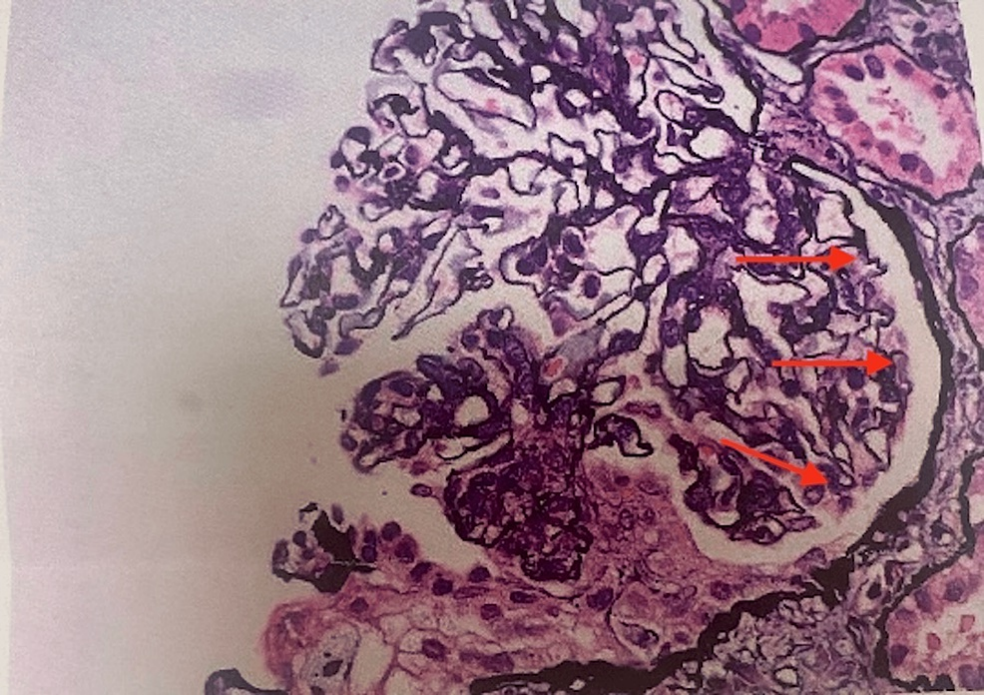
Kidney biopsy demonstrating crescent formation (red arrows)

**Figure 5:**
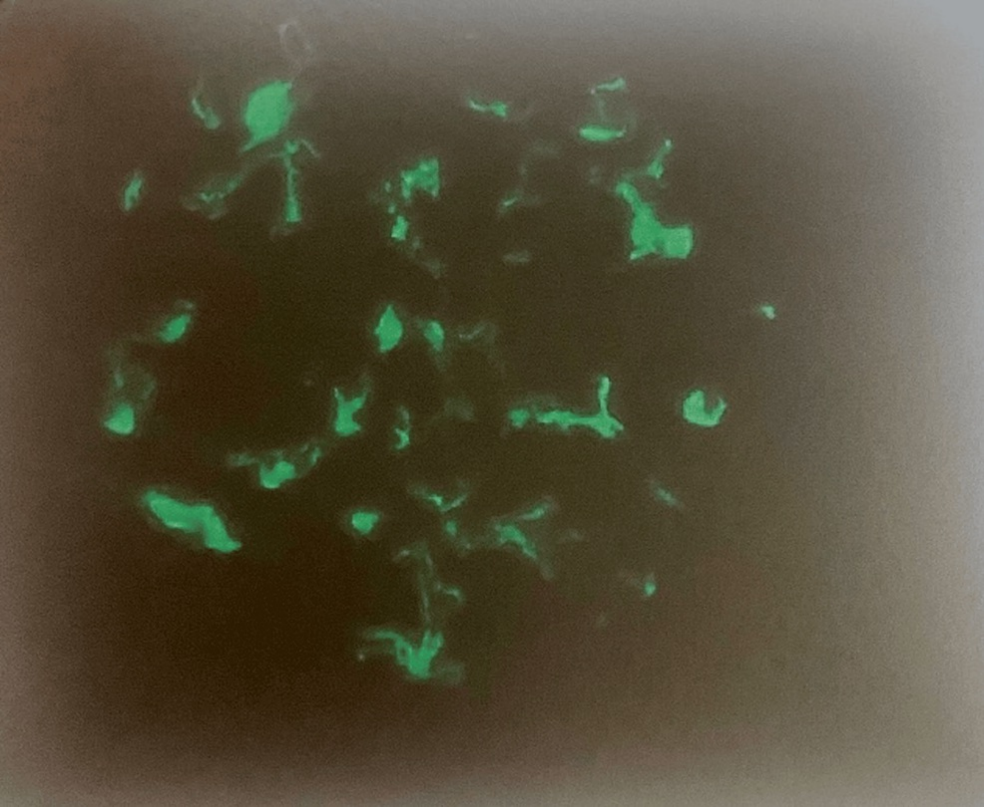
Kidney immunofluorescence image demonstrating IgA deposits

The patient was seen by dermatology as an outpatient, and a skin biopsy showed IgA vasculitis and she was re-started on prednisone. A kidney biopsy was performed, which confirmed the diagnosis of IgA nephropathy with focal crescents, mild to moderate activity, and mild chronicity (Figures 2-5).

It also showed mild tubular atrophy, interstitial fibrosis, and mild arterio- and arteriolosclerosis.

The patient had no family history of vasculitis or autoimmune disease. Immunological work-up was negative for SS-A, SS-B, P-ANCA, C-ANCA, GBM antibody, and DS-DNA. ACE, C4, and C3 were all within normal limits. ANA was low positive at 1:80 with a homogeneous pattern and IgA was increased at 383 mg/dL. She was eventually diagnosed with fibromyalgia and started on Cymbalta with a good response.

The patient was started on high-dose steroids, losartan, calcium, and vitamin D by a nephrologist for IgA nephropathy. Allergy testing later confirmed that the patient was not allergic to shellfish; therefore, no likely trigger was ever identified. IgA vasculitis signs and symptoms have completely resolved. The patient was managed for the IgA nephropathy on a steroid taper and responded well to treatment. On follow-up, the patient is following up with rheumatology and is stable on mycophenolate 1000 mg twice daily, losartan 25 mg daily, and prednisone 10 mg daily, and her proteinuria has decreased significantly.

## Discussion

IgA vasculitis, also known as Henoch-Schönlein purpura, is an inflammatory condition characterized by inflammation of small vessels secondary to perivascular deposition of IgA and activation of neutrophils [1]. It is characterized by the combination of palpable purpura, GI symptoms, and arthralgia. The prevalence of renal involvement in children and adults with IgA vasculitis is 20-54% and 45-80%, respectively [3]. Most commonly, this condition presents in children and has an identifiable trigger such as a GI or upper respiratory infection, medications, insect bites, and certain vaccinations [9]. Rarely, IgA vasculitis can present subsequent to an allergic reaction to food [10-12]. However, allergy testing confirmed that the described patient was not allergic to shellfish; thus, this was not a likely trigger. Thus, this case is unique because IgA vasculitis with no identifiable trigger presented in an adult, which is quite rare.

There are no specific criteria for diagnosing IgA vasculitis in adults, although the European Alliance of Associations for Rheumatology criteria for diagnosing IgA vasculitis in the pediatric population is often useful in aiding diagnosis in the adult population. Definitive diagnosis requires a biopsy of the lesions which usually reveal leukocytoclastic vasculitis affecting superficial vessels with predominantly IgA deposits or a kidney biopsy revealing proliferative glomerulonephritis with predominant IgA deposits. Renal complications are seen typically a few weeks after the initial presentation. Common presentations include proteinuria (>0.3 g/24 h or 30 mg/mmol), microscopic or macroscopic hematuria with or without an RBC cast, and hypertension [5]. Chronic kidney disease is a long-term complication of IgA vasculitis in the adult population. The adult population has also been shown to have a more aggressive disease course compared to the pediatric population and, therefore, will often benefit from a more intense treatment regimen. Early diagnosis and management are crucial in preventing long-term complications. Mild to moderate cutaneous and articular manifestations are symptomatically managed with rest, elevation, and pain management. Nonsteroidal anti-inflammatory drugs are usually avoided for analgesia considering the renal complications associated with IgA vasculitis. Refractory cases can be managed with oral steroids. As per the Kidney Disease Improving Global Outcomes guidelines, cyclophosphamide and steroids are used as treatment agents in the induction phase of IgA nephritis treatment. Certain trials have shown that using mycophenolate mofetil as a steroid-sparing agent may also be beneficial. GI manifestations are due to bowel ischemia and edema in the setting of vasculitis and can mimic the presentation of inflammatory bowel disease. These cases are also treated with IV steroids and cyclophosphamide, although severe cases with bowel perforation or infarction require surgical intervention [5,13]. Generally, adults affected by IgA vasculitis experience a more severe progression of the disease and often require more intense treatment approaches. Prompt diagnosis and timely treatment have demonstrated positive outcomes in managing renal complications linked to the disease.

The patient also soon after developed IgA nephropathy. IgA nephropathy is characterized by IgA deposits in the glomerular mesangium, often leading to macroscopic hematuria [2]. This condition most commonly develops in adulthood, usually during the late teenage years to late thirties [2]. A major risk of IgA nephropathy is that it can progress to end-stage renal disease, requiring patients to be on dialysis, and this occurs in approximately 30-40% of adults within 10-25 years from initial diagnosis [3]. Thus, when IgA nephropathy is suspected, vigilant monitoring of patients is required so that a kidney biopsy can be performed if necessary to diagnose the condition and enable treatment initiation as soon as possible.

While the pathophysiology of both IgA vasculitis and IgA nephropathy involves IgA deposition, there is no true consensus supported by clinical trials regarding whether these are different clinical manifestations of the same disease that occur on a spectrum, or if these are separate conditions, which may explain why many patients with IgA nephropathy never develop IgA vasculitis and vice versa [3]. In fact, the frequency of developing both of these conditions at the same time, one condition directly after the other, as in the described patient, and one condition long after the other has, to our knowledge, not been investigated by any clinical trials to date. This is where far more research is needed in order to identify patterns that may explain why these conditions remain isolated in certain patients, patients that are at the highest risk for developing both of these conditions, and, ultimately, whether they truly are different manifestations of the same condition or separate entities, as their unique treatment regimens may suggest.

## Conclusions

This case is distinctive because two clinical manifestations of IgA deposition, one involving cutaneous vessels and the other involving renal vessels, developed in an adult with no identifiable trigger. This series of events is so rare that, to our knowledge, despite few documented cases in the literature, there are no formal trials investigating the frequency of both presentations occurring together and/or one after the other. Given that this patient made several trips to the emergency department before being correctly diagnosed, this case not only highlights the importance of ruling out these conditions, especially IgA vasculitis, in adults presenting with a petechial rash but also the need for studies that investigate whether these conditions are related, how often these manifestations (and possible others) occur together, and what the predisposing factors are so that patients can be appropriately diagnosed and treated as efficiently as possible.
